# *De novo* genome assembly of two tomato ancestors, *Solanum pimpinellifolium* and *Solanum  lycopersicum* var. *cerasiforme*, by long-read sequencing

**DOI:** 10.1093/dnares/dsaa029

**Published:** 2021-01-19

**Authors:** Hitomi Takei, Kenta Shirasawa, Kosuke Kuwabara, Atsushi Toyoda, Yuma Matsuzawa, Shinji Iioka, Tohru Ariizumi

**Affiliations:** 1 Graduate School of Life and Environmental Sciences, University of Tsukuba, Tsukuba 305-8572, Japan; 2 Research Fellow of Japan Society for Promotion of Science (JSPS), Kojimachi, Tokyo 102-0083, Japan; 3 Department of Frontier Research and Development, Kazusa DNA Research Institute, Kisarazu, Chiba 292-0818, Japan; 4 Comparative Genomics Laboratory, Advanced Genomics Center, National Institute of Genetics, Mishima, Shizuoka 411-8540, Japan; 5 Comparative Genomics Laboratory, National Institute of Genetics, Mishima, Shizuoka 411-8540, Japan; 6 TOKITA Seed Co. LTD, Otone, Saitama 349-1144, Japan; 7 Tsukuba Plant Innovation Research Center, University of Tsukuba, Tsukuba, Ibaraki, 305-8572, Japan; 8 Faculty of Life and Environmental Sciences, University of Tsukuba, Tsukuba, Ibaraki 305-8572, Japan

**Keywords:** whole-genome comparison, gene annotation, long-read sequencing, wild tomato, RNA-Seq

## Abstract

The ancestral tomato species are known to possess genes that are valuable for improving traits in breeding. Here, we aimed to construct high-quality *de novo* genome assemblies of *Solanum pimpinellifolium* ‘LA1670’ and *S. lycopersicum* var. *cerasiforme* ‘LA1673’, originating from Peru. The Pacific Biosciences (PacBio) long-read sequences with 110× and 104× coverages were assembled and polished to generate 244 and 202 contigs spanning 808.8 Mbp for ‘LA1670’ and 804.5 Mbp for ‘LA1673’, respectively. After chromosome-level scaffolding with reference guiding, 14 scaffold sequences corresponding to 12 tomato chromosomes and 2 unassigned sequences were constructed. High-quality genome assemblies were confirmed using the Benchmarking Universal Single-Copy Orthologs and long terminal repeat assembly index. The protein-coding sequences were then predicted, and their transcriptomes were confirmed. The *de novo* assembled genomes of *S. pimpinellifolium* and *S. lycopersicum* var. *cerasiforme* were predicted to have 71,945 and 75,230 protein-coding genes, including 29,629 and 29,185 non-redundant genes, respectively, as supported by the transcriptome analysis results. The chromosome-level genome assemblies coupled with transcriptome data sets of the two accessions would be valuable for gaining insights into tomato domestication and understanding genome-scale breeding.

## 1. Introduction

Plant domestication and improvements enable the selection of genes and alleles that are favourable to humans, whereas numerous genes/alleles that are unfavourable to humans are lost in the process. Some of these losses have proved undesirable for breeding. Cultivated tomato [*Solanum lycopersicum* var. *lycopersicum* (SLL)], which originates from South America, likely occurred in both Peru and Ecuador.^1^ Cultivated tomatoes have become one of the most important vegetable crops worldwide; they are a rich source of vitamins, minerals, and fibre, as well as a dietary source of antioxidants, although some valuable genes (e.g. a subset of genes associated with biotic and abiotic stresses) were likely lost as an unwanted effect of domestication and improvements.^2–5^ Due to its global nutritional and economical importance, whole-genome sequence (WGS) of a domesticated tomato accession ‘Heinz 1706 (LA4345)’ was determined in 2012, and it has been updated to date, serving as a valuable resource in both basic and applied research.^6^^,^^7^ On the contrary, ancestral species close to cultivated accessions carry genetic potentials that exert favourable traits. For example, some accessions of *S. pimpinellifolium* (SP), known to be ancestral tomatoes, exhibit higher tolerance to salt stress and tomato yellow leaf curl virus.^8–10^ Furthermore, *S. lycopersicum* var. *cerasiforme* (SLC), which originated from SP, is also expected to be an ancestor of domesticated tomato and possesses rich genetic loci contributing to increased fruit size.^11^^,^^12^ Additionally, genome-wide association studies of 163 accessions within these *Solanum* species have revealed a large extent of morphological variations in their flowers and fruit and high intraspecific genome diversity and interspecific diversity.^1^ Recently, tomato pan-genome analyses have revealed several presence/absence variations within both inter- and intra-genic regions, and they are likely associated with fruit and flower morphological variations and contribute to improve traits.^13^^,^^14^ Thus, increasing available genome information of these ancestral species will facilitate the identification of unique or novel genes that have been lost during the breeding processes.

To obtain WGS information, ‘LA1589’ was used to obtain the first draft genome of SP,^6^ and genome assembly and annotation of ‘LA0480’ were achieved using 101 bp paired-end (PE) short-read libraries and 5 mate-pair libraries. However, both data sets are highly fragmented and incomplete.^15^ Recently, chromosome-scale genome sequence of ‘LA2093’ was determined using a long-read PacBio sequencer and was annotated to contain 35,761 protein-coding genes, of which 35,535 were validated by the published transcriptome data and/or homologs in the NCBI non-redundant protein database.^16^ Furthermore, draft genome sequences of several SP and SLC accessions have been determined using a long-read Nanopore sequencer, and several structural variant hotspots have been identified.^14^ However, chromosome-level genome assemblies, particularly those generated using long-read sequencers, are still limited and should be further improved. In this study, we newly constructed genome assemblies of two ancestral accessions, SP accession ‘LA1670’ and SLC accession ‘LA1673’, which originated in Tana and Lima, Peru, respectively. We first assembled long-read sequences generated using the PacBio Sequel system, and they were polished with Illumina short reads to generate chromosome-level scaffolds. Over 70,000 protein-coding sequences were annotated in both accessions and transcriptome datasets supported the mRNA expression for approximately 39% of the annotated genes. The newly constructed genome assemblies of tomato ancestors will be valuable to explore interspecific and intraspecific variations as well as to identify genes that could improve traits in breeding.

## 2. Materials and methods

### 2.1 Processing data for the WGS of tomato

**Figure 1:**
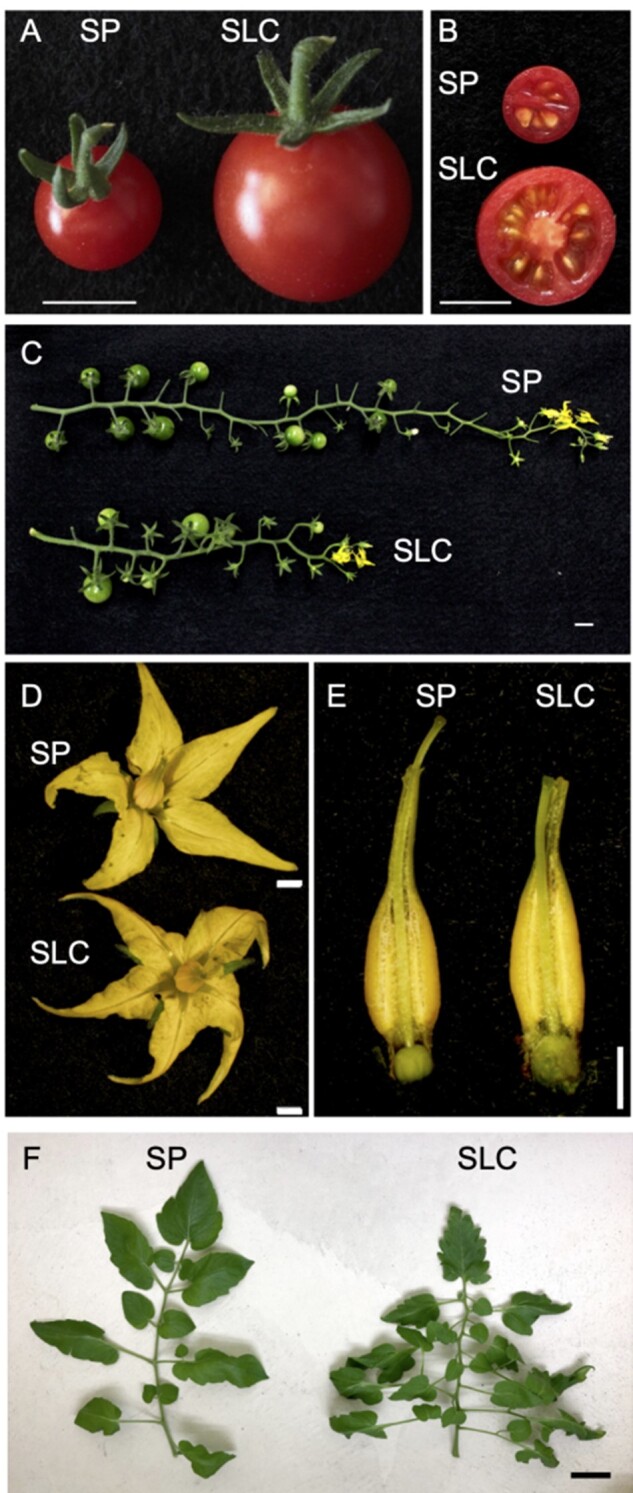
Morphology of S. pimpinellifolium ‘LA1670’ and Solanum lycopersicum var. cerasiforme ‘LA1673’. Morphology of (A) fruit, (B) cross section of fruit, (C) inflorescence, (D) flower, (E) pistil and stamen, and (F) mature leaves of SP and SLC. SP, *S. pimpinellifolium*; SLC, *S. lycopersicum* var*. cerasiforme.* Bars are 1 cm (A, B, C); 200 μm (D, E), 5 cm (F).

DNA from young leaves of ‘LA1670’ and ‘LA1673’ (Fig. 1 and Supplementary Fig. S1) was extracted from a single plant of each line using the Maxwell 16 Cell DNA Purification Kit (Promega, Madison, WI, USA) and used for Illumina sequencing. Sequencing data were obtained using Illumina HiSeq 2000 (Illumina, San Diego, CA, USA) with a 150 bp PE mode. Sequenced bases with a quality score of <30 and adaptor sequences were removed using TrimGalore (version 0.6.4) with option –length 90. The quality of the trimmed sequences was checked using FastQC. SMRT sequence libraries were constructed using the SMRTbell Express Template Prep Kit (PacBio, Menlo Park, CA, USA) and used to sequence on a PacBio Sequel system (PacBio, Menlo Park, CA, USA). The long-read sequences were assembled, and the two haplotype sequences of the diploid genome were resolved using Falcon-unzip. Errors in the resultant assembled data were corrected twice by Arrow and polished with 47× Illumina PE data using Pilon, which includes correcting bases, fixing misassemblies, and filling gaps. Chromosome-scale pseudomolecules were produced using reference-guided scaffolding software RaGOO. The pseudomolecule sequences of SP ‘LA1670’ (SPI_r1.0 pmol) and SLC ‘LA1673’ (SLYcer_r1.0 pmol) were named from SPI1.1ch00 to SPI1.1ch12 and from SLYcer1.1ch00 to SLYcer1.1ch12, respectively, according to the mapped chromosomes from SL4.0ch00 to SL4.0ch12 in the SLL ‘Heinz’ genome assemblies (SL4.0). The remaining unassigned sequences of SP and SLC were grouped into SPIUN and SLYcerUN, respectively (UN, unknown). The genome structure of ‘LA1670’ (SPI_r1.0 pmol) and ‘LA1673’ (SLYcer_r1.0 pmol) were compared against ‘LA4345’ (SL4.0, Heinz 1706) using D‐GENIES with Minimap2 aligner, and resultant genome assemble of SP and SLC were named as SPI_r1.1 pmol and SLYcer_r1.1 pmol.

To estimate the genome size, *k*-mer counting analysis was performed using jellyfish software as described previously.^17^ The software and related references used in this study are shown in Supplementary Table S1.

### 2.2 Assessing assembly completeness

We first assessed the completeness of ‘LA1670’ and ‘LA1673’ genome assemblies using the Benchmarking Universal Single-Copy Orthologs (BUSCO) database (v.4.1.1) in the genome mode to search for genes conserved in Embryophyta species. The embryophyta_odb10 data set, created on 20 November 2019, comprises 57 species and 425 genes. The genome completeness of SP and SLC was further analysed using long terminal region (LTR) assessing index (LAI), which is used to evaluate genome quality based on intergenic genome information using LTR retrotransposon (LTR-RT).^18^ LTR-RT candidates were obtained using LTRharvest and LTR_FINDER_parallel and their accurate LTR-RTs were determined using LTR_retriever as described previously.^18^ The accurate LTR-RTs were then used to evaluate assembly continuity of the input genome using the LAI with default parameters.

### 2.3 Repetitive sequence detection

Repetitive sequences were detected using RepeatMasker v.4.0.7, where we used repeat sequences obtained from the pseudomolecule sequences, SPI_r1.1 pmol and SLYcer_r1.1 pmol, using RepeatModeler v.1.0.11 and a dataset registered in Repbase.

### 2.4 Gene annotation validation with RNA-Seq

Putative protein‐coding genes in the genome sequences were predicted using MAKER pipeline, for which we used the RNA-Seq data and peptide sequences predicted from genomes of a tomato (SLL, ITAG4.0), potato (SolTub_3.0), and pepper (ASM51225v2). Two training sets from AUGUSTUS and SNAP were also used in the prediction. Sequences with an annotation edit distance score of >0.5 were selected as high-confidence genes.

The total RNA was extracted from 17 organ samples consisting of seven anther samples of different developmental stages (bud size: 3–4, 4–5, 5–6, 6–7, 7–8, and 8–9 mm, and anthesis), anthesis pollen, calyx, green fruit, red fruit, leaf, ovary, white petal of a flower 1-day before anthesis, petal of an anthesis flower, root, and stem from hydroponic-cultivated plants of ‘LA1670’ and ‘LA1673’. The plants were grown in a greenhouse at the University of Tsukuba, Japan. All organs were sampled in the morning between December 2018 and January 2019, and RNA was extracted using RNeasy Mini Kit, and then purified using RNase-Free DNase Set (QIAGEN, USA). Genome-wide RNA expression levels were obtained using Novaseq-PE150 (Illumina) at a depth of 78 Gbp reads. The RNA-Seq data were first trimmed; the bases with a quality score of <30 and adapters were removed using TrimGalore with the option –length 90. We filtered ribosomal RNA (rRNA) reads by applying SortMeRNA. For Iso-Seq analysis, the total RNA was purified as described above and used for cDNA synthesis and amplification using the NEBNext Single Cell/Low Input cDNA Synthesis & Amplification Module and Iso-Seq Express Oligo Kit. A sequencing library was constructed using the SMRTbell Express Template Prep Kit 2.0 and was run on a PacBio Sequel system using the Sequel Binding Kit 3.0/Sequel Sequencing Kit 3.0 according to the manufacturer’s protocol.

### 2.5 Estimating gene and isoform expression levels from RNA-Seq data

The cleaned reads, devoid of rRNA, were mapped against the assembled genomes and the transcription levels of each gene were calculated using STAR-RSEM pipeline. Genes with transcripts per million (TPM) values of >0 were regarded as expressed. After filtering genes whose expression levels were zero in all tissue samples, the expressed genes were counted using an in-house python script. The transcripts whose isoforms were identified by full-length Iso-Seq sequencing were also regarded as expressed genes.

## 3. Results and discussion

### 3.1 Overview of the two unimproved tomato assembly process

**Figure 2:**
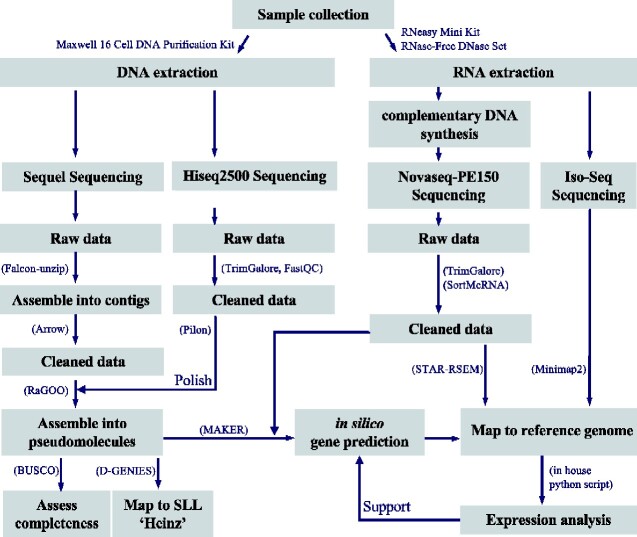
Work-flow used in this study for constructing genomes.

Figure 2 represents the experimental design and pipelines used in this study. The genome information of ‘LA1670’ and ‘LA1673’ (Fig. 1 and Supplementary Fig. S1) were obtained from PacBio Sequel and the Illumina HiSeq 2000 sequencing platforms, resulting in 86.8 Gb (PacBio) and 112.4 Gb (Illumina), and 77.6 Gb (PacBio) and 110.8 Gb (Illumina) data, respectively. After removing low-quality data and adopter sequences from Illumina PE data, 79.7 and 78.1 Gb sequence data remained for SP and SLC, respectively (Supplementary Tables S2 and S3). The obtained PacBio data of SP and SLC were then assembled into 244 and 201 primary contigs. To correct sequencing errors caused by PacBio sequencers, these contigs were polished using the 47× coverage of cleaned Illumina PE reads with Pilon. The resultant error-corrected contig sequences were 808.8 Mbp with contig N50 of 10.12 Mbp for SP and 804.5 Mbp with N50 of 9.48 Mbp for SLC; these contig sets were named as SPI_r1.1, SLC_r1.1, respectively (Table 1). These assembly sizes were almost equivalent to 786.2 and 770.4 Mbp of genome sizes estimated using the *k*-mer counting analyses (*k*-mer = 21; Supplementary Fig. S2) and were comparable size of the SLL reference ‘LA4345’ (SL4.0; 782,520,133).

**Table 1:** Statistics of final assembly of *S. pimpinellifolium* ‘LA1670’ and *S. lycopersicum var. cerasiforme* ‘LA1673’ by PacBio Sequel system

	*S. pimpinellifolium* ‘LA1670’ (SPI_r1.1)^a^	*S. lycopersicum var. cerasiforme* ‘LA1673’ (SLYcer_r1.1)^b^
N50 (bp)	10,119,361	9,478,482
L50	24	22
N60 (bp)	8,226,607	8,392,046
L60	33	31
N70 (bp)	6,166,144	6,753,289
L70	44	41
N80 (bp)	4,109,631	4,680,801
L80	60	55
N90 (bp)	2,273,512	2,681,313
L90	86	77
Number of contigs	244	202
Largest contig (bp)	36,305,789	44,614,784
Smallest contig (bp)	21,255	23,004
Average contig length (bp)	3,314,667	3,982,507
Total length (bp)	808,778,754	804,466,445

a DDBJ accession numbers: BMBV01000001–BMBV01000244.

b DDBJ accession numbers: BMBW01000001–BMBW01000202.

### 3.2 Comparison of genome assemblies

**Figure 3:**
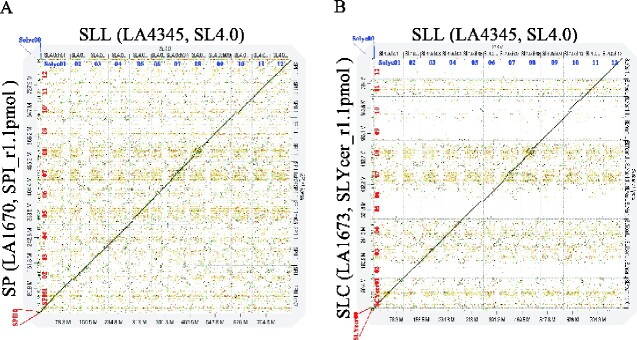
Dot plot analysis of the alignments between the constructed genomes and *S. lycopersicum* genome assemblies. Genomes of SP ‘LA1670’ (SPI_r1.1pmol) (A) and SLC ‘LA1673’ (SLYcer_r1.1pmol) (B) were mapped to SLL ‘Heinz’ (SL4.0). Each chromosome name (from ch00 to ch12) refers from SPI00 to SPI12 in SP ‘LA1670’ and from SLYcer00 to SLYcer12 SLC ‘LA1673’, and they were assigned from Solyc00 to Solyc12 in the reference genome of SLL ‘Heinz1706’ (SL4.0). Unknown refers to the unassigned contigs of SP ‘LA1670’ or SLC ‘LA1673’. SP, *S. pimpinellifolium*; SLC, *S. lycopersicum* var*. cerasiforme*; SLL, *S. lycopersicum* var*. lycopersicum.*

Reference-guided scaffolding of the error-corrected contigs was performed using RaGOO combined with the SLL (SL4.0), which included the tomato 12 chromosomes (ch01–ch12) and unanchored genome sequences designated ch00 that are not mapped to any of the 12 chromosomes. Polished contigs were ordered and orientated using the SL4.0 reference. Then, 242 and 200 contigs of SP and SLC, respectively, were mapped to the 12 tomato chromosomes (SL4.0ch01–ch12; Table 2). Furthermore, two contigs consisting of 1,449,357 bp from SP and 61,018 bp from SLC were non-localized to the corresponding reference genome and assigned as ‘unknown’ (SPIUN and SLYcerUN). These unassigned ‘unknown’ contigs were considered as genomic segments specifically present in SP and SLC. Finally, RaGOO analysis resulted in the total scaffold lengths of 808.8 and 804.5 Mbp, and average scaffold length of 57.8 and 57.5 Mbp, respectively, for SP and SLC (Table 3). These chromosome-level assemblies of SP and SLY were named SPI_r1.1 pmol and SLYcer_r1.1 pmol, respectively (Table 3). The dot plot analysis using D-GENIES between SLL (SL4.0) and both SP and SLC revealed significant consistency for genome organization, while identifying potential structural variants (i.e. indels and inversions) between these species (Fig. 3).

**Table 2:** Statistic of the *S. pimpinellifolium* ‘LA1670’ and *S. lycopersicum* var. *cerasiforme* ‘LA1673’ pseudomolecule sequences

	*S. pimpinellifolium* ‘LA1670’						*S. lycopersicum* var. *cerasiforme* ‘LA1673’						*S. lycopersicum* var. *lycopersicum* ‘Heinz1706’ (SL4.0_ITAG4.0)			
Chromosome^a^	Contigs^b^	(%)	Contig size(bp)	(%)	Genes^c^	(%)	Contigs^b^	(%)	Contig size(bp)	(%)	Genes^c^	(%)	Sequence length(bp)	(%)	Genes^c^	(%)
Ch00	32	13.1	6,197,042	0.8	4,544	6.3	37	18.3	9,678,733	1.2	6,727	8.9	9,643,250	1.2	513	1.5
Ch01	18	7.4	97,789,368	12.1	8,859	12.3	11	5.4	94,371,439	11.7	8,618	11.5	90,863,682	11.6	4,133	12.1
Ch02	14	5.7	49,861,979	6.2	4,789	6.7	8	4.0	54,091,648	6.7	5,863	7.8	53,473,368	6.8	3,379	9.9
Ch03	20	8.2	65,162,952	8.1	5,662	7.9	17	8.4	66,823,253	8.3	5,558	7.4	65,298,490	8.3	3,324	9.8
Ch04	9	3.7	65,616,834	8.1	5,625	7.8	13	6.4	67,222,826	8.4	6,029	8.0	64,459,972	8.2	2,819	8.3
Ch05	24	9.8	75,079,728	9.3	5,018	7.0	10	5.0	57,125,772	7.1	3,376	4.5	65,269,487	8.3	2,382	7.0
Ch06	9	3.7	49,929,902	6.2	5,439	7.6	10	5.0	48,343,254	6.0	5,578	7.4	47,258,699	6.0	2,769	8.1
Ch07	23	9.4	74,519,832	9.2	6,430	8.9	23	11.4	72,923,569	9.1	5,786	7.7	67,883,646	8.7	2,517	7.4
Ch08	11	4.5	68,510,109	8.5	6,471	9.0	7	3.5	70,551,588	8.8	6,557	8.7	63,995,357	8.2	2,428	7.1
Ch09	24	9.8	71,053,701	8.8	5,001	7.0	15	7.4	68,740,419	8.5	5,308	7.1	68,513,564	8.8	2,521	7.4
Ch10	12	4.9	60,610,119	7.5	4,537	6.3	14	6.9	72,371,102	9.0	6,070	8.1	64,792,705	8.3	2,520	7.4
Ch11	12	4.9	56,468,337	7.0	4,336	6.0	19	9.4	55,383,786	6.9	4,268	5.7	54,379,777	6.9	2,326	6.8
Ch12	34	13.9	66,552,494	8.2	5,186	7.2	16	7.9	66,796,838	8.3	5,274	7.0	66,688,036	8.5	2,444	7.2
Unknown	2	0.8	1,449,357	0.2	48	0.1	2	1.0	61,018	0.0	218	0.3		0.0		0.0
Total	244	100	808,801,754	100	71,945	100	202	100	804,485,245	100	75,230	100	782,520,033	100	34,075	100

a In the Chromosome column, each chromosome name (from Ch00 to Ch12) refers from SPI00 to SPI12 in *S. pimpinellifolium* ‘LA1670’ and from SLYcer00 to SLYcer12 in *S. lycopersicum* var. *cerasiforme* ‘LA1673’ which assigned to from Solyc00 to Solyc12 of the reference genome of *S. lycopersicum* var. *lycopersicum* ‘Heinz1706’ (SL4.0). Unknown refers to the unassigned contigs of *S. pimpinellifolium* ‘LA1670’ or *S. lycopersicum* var. *cerasiforme* ‘LA1673’.

b contig, the number of contigs mapped on each chromosome.

c Gene, the number of genes annotated on each chromosome.

**Table 3:** Scaffolding statistics of *S. pimpinellifolium* ‘LA1670’ and *S. lycopersicum* var. *cerasiforme* ‘LA1673’ after RaGOO and D-genies analyses

Statistics	*S. pimpinellifolium ‘*LA1670’	*S. lycopersicum* var*. cerasiforme ‘*LA1673’
N50 (bp)	66,552,494	66,823,253
L50	6	6
N90 (bp)	49,929,902	48,343,254
L90	11	11
Number of scaffolds	14	14
Largest scaffold (bp)	97,789,368 (SPI1.1ch01)	118,650,774 (SLYcer1.1ch01)
Smallest scaffold (bp)	1,449,357 (SPI1.1Unknown)	61,018 (SLYcer1.1Unknown)
Average scafold length (bp)	57,771,554	57,463,225
Total length (bp)	808,801,754	804,485,145

Statistics for the genome version SPI_r1.1 pmol and SLYcer_r1.1 pmol were shown.

### 3.3 Estimation of assembly completeness

The completeness of the genome assembly for all scaffolds and non-localized contigs were quantified using the BUSCO database. We used the embryophyta_odb10 data set containing 1,624 core genes. Scaffolds and non-localized contigs of SP contained a higher number of benchmarking genes (96.2%), and a few of those (1.4%) were duplicated as isoform genes according to our BUSCO analyses (Table 4). In contrast, 96.5% of the expected Embryophyta genes were detected as complete BUSCOs in the scaffolded genome sequence of SLC. The benchmarking gene preservation of SP and SLC was almost equivalent to that of SLL (SL4.0; 97.5%).^19^

**Table 4:** Statistics of the polished scaffold sequences of *S. pimpinellifolium ‘*LA1670’ and *S. lycopersicum* var. *cerasiforme* ‘LA1673’

	*S. pimpinellifolium* ‘LA1670’	*S. lycopersicum* var*. cerasiforme* ‘LA1673’
Complete BUSCOs (%)	96.2	96.5
Single-copy BUSCOs (%)	94.8	95.1
Duplicated BUSCOs (%)	1.4	1.4
Fragmented BUSCOs (%)	1.2	1.1
Missing BUSCOs (%)	2.6	2.4
Total BUSCO groups	1,614	1,614

We used the Benchmarking Universal Single-Copy Orthologs (BUSCO) (v. 4) method with Embryophyta core gene dataset to evaluate the completeness of the genome annotation.

The completeness of these genome assemblies and that of SLL (SL4.0) were further validated using LAI, which is used to evaluate assembly continuity and quality of intergenic and repetitive sequences by counting the proportion of intact LTR-RTs in the assembled genome. A higher LAI score is associated with a higher assembly quality of repetitive and intergenic sequence spaces, because of the likely identification of a higher number of intact LTR-RTs.^18^ The overall distribution of LAI scores was similar among three accessions (Supplementary Fig. S3), but the whole genome LAI score of SLL (SL4.0) was higher (LAI = 10.37) than that of SLL SL2.4 (LAI = 8.0),^19^ which were sequenced by short reads. Interestingly, SP and SLC presented relatively higher LAI scores, 14.18 and 13.10, respectively. Wild tomato species most likely have a higher number of identifiable intact LTRs and/or longer internal regions than cultivated species, as the LAI score of other SP (LA2079; LAI = 13.14) and *S. pennellii* (LAI = 14.8) is higher than that of SLL.^16^^,^^18^ According to these quality assessment results, the SP and SLC genomes were classified to be of reference quality based on the assembly of repetitive and intergenic sequence spaces (draft quality, LAI < 10; reference quality, 10 < LAI < 20; and gold quality LAI > 20).^19^

We then analysed repetitive and transposal sequences in SP (SPI_r1.1 pmol) and SLC (SLYcer_r1.1 pmol). The repetitive sequences comprised 535.3 Mb (66.2%) and 532.6 Mb (66.2%) pseudomolecule sequences of SLC and SP, respectively (Supplementary Table S4). The dominant types in the pseudomolecule sequences were LTR-RT (409.7 Mb for SP and 402.0 Mb for SLC), followed by DNA transposons. Specific repeat sequences that were currently unavailable in public databases were 54.3 Mb for SP and 55.3 Mb for SLC. These results indicated that both SP and SLC contain a similar proportion of repetitive and transposal sequences.

**Figure 4:**
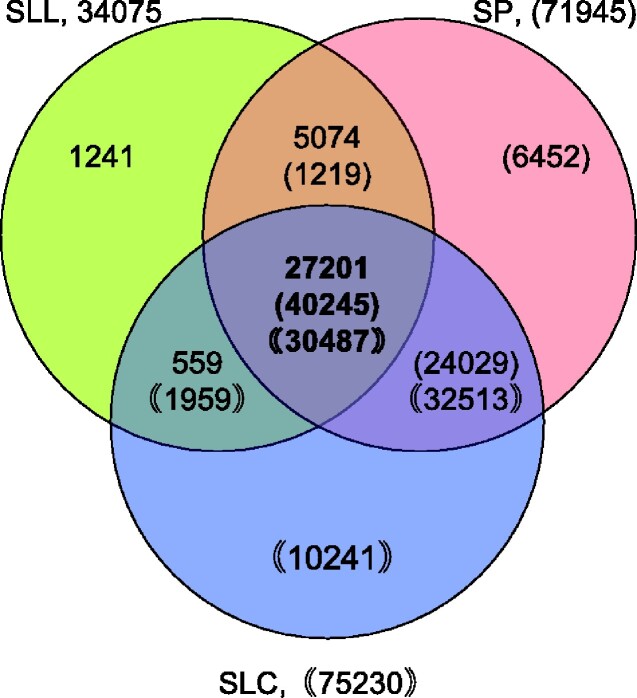
Number of genes commonly identified among the reference and ancestral tomatoes. Venn graph shows the comparison of redundant and unique genes among the reference tomato (ITAG4.0, SLL_ITAG4.0), SP (SPI_r1.1pmol), and SLC (SLYcer_r1.1pmol). Total gene numbers for SLL, SP, and SLC were 34,075, 71,945, and 75,230, respectively. The number of genes that were common exited among three tomatoes was 27,201, 40,245, and 30,487, in ITAG4.0, SP, and SLC, respectively. The number of genes found in only the reference tomato genes, SP, and SLC were 1,241, 6,452, and 10,241, respectively. The number of total genes for SLL is shown as numbers without brackets, whereas those for SP and SLC are shown as numbers in single and double brackets, respectively. SP, *S. pimpinellifolium*; SLC, *S. lycopersicum* var. *cerasiforme*; SLL, *S. lycopersicum* var. *lycopersicum*.

Next, putative protein-coding genes in the constructed SP (808.8 Mbp) and SLC (804.5 Mbp) genome assemblies were predicted using MAKER pipeline. As a result, 71,945 and 75,230 potential coding sequences were predicted, respectively (Table 2). We then analysed the number of genes commonly or uniquely present in the reference SLL (ITAG4.0), SP, and SLC. Among 34,075 SLL genes, 32,275 (27,201 + 5,074) and 27,760 (27,201 + 559) homologous genes existed in SP and SLC, respectively (Fig. 4). Furthermore, 27,201 SLL genes (79.8%) were present in these three genotypes, with 40,245 and 30,487 genes in SP and SLC, respectively. The fact that 94.7% (32,275/34,075) and 81.5% (27,760/34,075) of SLL genes were present in SP and SLC, respectively, suggests that the genome of ‘LA1670’ is more closely related to ‘LA4345’ than to ‘LA1673’.

### 3.4 Expression gene analyses

Together with the Illumina RNA-Seq and Iso-Seq reads, 29,629 and 29,185 non-redundant protein-coding sequences for SP and SLC were supported by transcriptome datasets, respectively (Table 5). We obtained RNA-Seq Illumina reads derived from 17 samples of each line to validate gene prediction. All raw reads were trimmed using TrimGalore, which generated 79.7 and 78.1 Gb of high-quality data with a high mapping rate (>96%) from 112.4 and 110.8 Gb of raw data in ‘LA1670’ and ‘LA1673’, respectively (Supplementary Tables S5 and S6). After removing rRNA contamination using SortMeRNA, the cleaned reads were mapped to each of the corresponding genome assemblies of SP (SPI_r1.1 pmol) and SLC (SLYcer_r1.1 pmol) using the STAR-RSEM pipeline, resulting in a mapping rate of 97.0% for SP and 97.8% for SLC. We regarded genes with a TPM value of >0 as expressed genes and identified 29,343 (SP) and 29,075 (SLC) expressed genes in the 17 samples. The protein-coding sequences were further validated by Iso-Seq to generate full-length cDNA sequences or isoforms. The Iso-Seq produced 38,569 and 27,302 isoforms with an average length of 2,657 and 2,428 bp from SP pollen and SLC mature anther, respectively (Supplementary Table S7). The isoform average size was approximately 12 kb with N50 of 2.6–2.8 kb. These isoforms were also mapped on the constructed genome assemblies using Minimap2, resulting in a high mapping rate of 99.98% for SP and 99.88% for SLC. Among the 71,945 SP and 75,230 SLC protein-coding sequences predicted from genome information, 8,296 and 4,135 of them were supported by Iso-Seq reads, respectively, and they were also regarded as expressed genes.

**Table 5:** Number of expressed genes supported by transcriptomes in *S. pimpinellifolium* ‘LA1670’ and *S. lycopersicum* var. *cerasiforme* ‘LA1673’

Chromosome	LA1670		LA1673	
	Genes^a^	(%)	Genes^a^	(%)
Ch00	20	0.1	37	0.1
Ch01	3,720	12.6	3,672	12.6
Ch02	2,906	9.8	2,896	9.9
Ch03	2,915	9.8	2,943	10.1
Ch04	2,528	8.5	2,467	8.5
Ch05	2,179	7.4	1,346	4.6
Ch06	2,537	8.6	2,505	8.6
Ch07	2,210	7.5	2,182	7.5
Ch08	2,174	7.3	2,101	7.2
Ch09	2,265	7.6	2,192	7.5
Ch10	2,102	7.1	2,836	9.7
Ch11	1,937	6.5	1,908	6.5
Ch12	2,129	7.2	2,100	7.2
Unknown	7	0.0		0.0
Total	29,629	100.0	29,185	100.0

Total number of expressed genes for SP was calculated as non-redundant genes that were supported by 29,343 illumina RNA-Seq reads and 8,296 Iso-Seq reads. Total number of expressed genes for SLC was calculated as non-redundant genes that were supported by 29,075 illumina RNA-Seq reads and 4,135 Iso-Seq reads.

a Gene, the number of genes annotated on each chromosome.

b (%), the proportion of gene numbers to the total gene number.

In conclusion, in this study, we constructed novel genome assemblies for two *Solanum* accessions, SP and SLC, using the PacBio long-read sequencer, and predicted their protein-coding sequences, 39% of which were supported by transcriptome datasets (Table 5). The genome size of SP and SLC was 808.8 and 804.5 Mbp, respectively (Table 3), which were almost equivalent to the genome sizes estimated using the *k*-mer counting analysis (Supplementary Fig. S2). Furthermore, using reference-guided scaffolding, we constructed 12 pseudomolecules at the chromosome level with the average scaffold length of 57.8 and 57.5 Mbp (Fig. 3, Table 3), which were considerably higher than that of the reference generated by short-read sequences (scaffold average length = ∼5 kbp).^6^^,^^15^ Besides, both SP and SLC genome assemblies presented high BUSCO completeness values (Table 4, 96.2% and 96.5%, respectively), and the LAI values were equivalent to another high-quality genomes, indicating the effectiveness of the long-read sequencing-based *de novo* assemble strategy.

SP has substantial intraspecific genome diversity because of the rich geographic and climatic varieties in their originated and migrated areas (e.g. Ecuador and Peru),^20–22^ whereas SLC shares SP genome structure as the most recent common ancestor with the cultivated SLL.^23^ Thus, our newly constructed genome assemblies will facilitate dissection of the genetic and molecular aspects of tomato domestication and evolution. For instance, SP ‘LA1670’ is known to show heat susceptibility,^24^ whereas SLC ‘LA1673’ shows salinity tolerance and accumulates higher levels of sugars, organic acids, and volatiles associated with tomato flavour than cultivated varieties.^25^^,^^26^ Moreover, in this study, we identified 6,452 and 10,241 unique genes from SP and SLC, respectively, which are not present in SLL (SL4.0; Fig. 4). Genome and gene information obtained in this study will be beneficial for dissecting these traits for breeding; it can also be used to explore gene function of these ancestral-specific genes.

## Supplementary data

Supplementary data are available at DNARES online.

## Supplementary Material

dsaa029_Supplementary_Data
