# Supplementary material for: De novo genome assembly of two tomato ancestors, Solanum pimpinellifolium and Solanum lycopersicum var. cerasiforme, by long-read sequencing
Source: DNA Res. 2021 Jan 19;28(1):dsaa029. doi: 10.1093/dnares/dsaa029 (PMC7934570; doi:10.1093/dnares/dsaa029)
Supplement: dsaa029_Supplementary_Data [file dsaa029_supplementary_data.zip › 20201207_Takei_et_al_Sup_Figures.pdf]

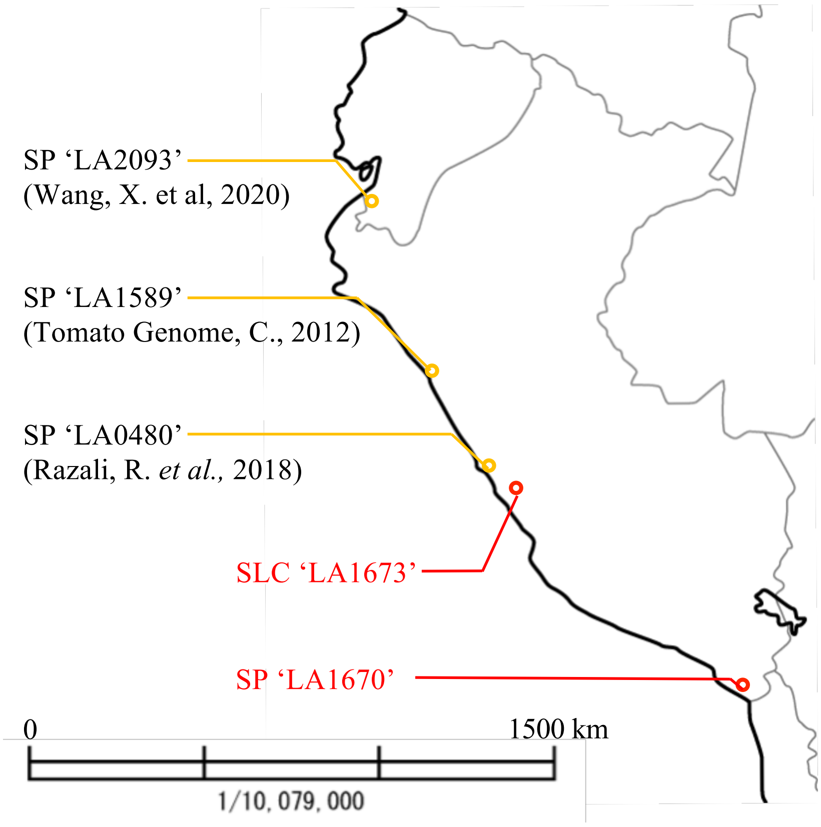

Supplementary Figure 1 Geographical location of accessions used in this study.

Origin of SP and SLC accessions used in this study are shown. Note that the origin of SP 'LA1670' was more southern than that of SP 'LA2093' and SP 'LA1589'. SP, *Solanum pimpinellifolium*, SLC, *Solanum lycopersicum* var. *cerasiforme.* Data courtesy of OpenStreetMap (openstreetmap.org; OpenStreetMap contributors).

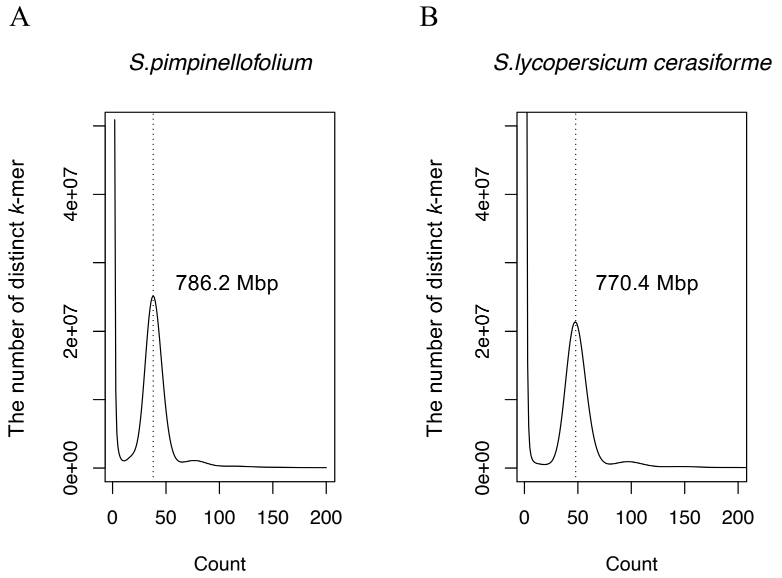

Supplementary Figure 2 Genome size estimation using the *k*-mer counting analysis.

Genome size of SLC 'LA1673' (A) and SP 'LA1670' (B) was estimated by the distribution of the number of distinct *k*-mers ($k = 21$) with the given multiplicity values. The X-axis indicates coverage *k*-mers. Dashed lines indicate the highest *k*-mer frequency for SP (*k*-mer = 48) and SLC (*k*-mer = 38). The Y-axis indicates the number of distinct *k*-mers. SP, *S. pimpinellifolium,* SLC, *S. lycopersicum cerasiforme*.

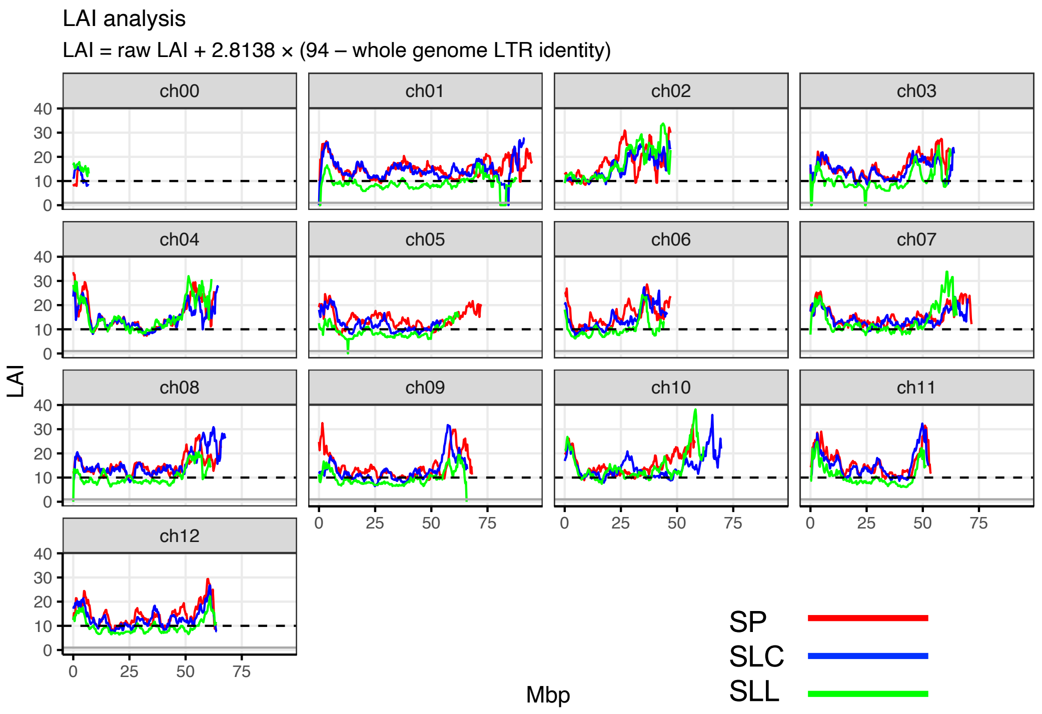

Supplementary Figure 3. Genome quality assessment by LAI analysis.
The quality and continuity of the assembled genomes of SP (SPI_r1.1pmol), and SLC (SLYcer_r1.1pmol) were evaluated by LAI method together with SLL (SL4.0). SP, *S. pimpinellifolium*; SLC, *S. lycopersicum* var. *cerasiforme*; SLL, *S. lycopersicum* var. *lycopersicum*; ch, chromosome; LTR, long terminal repeat; LAI, LTR Assembly Index.
